# Stigma and infectious diseases in Africa: examining impact and strategies for reduction

**DOI:** 10.1097/MS9.0000000000001470

**Published:** 2023-11-01

**Authors:** Pius Omoruyi Omosigho, Okesanya Olalekan John, Mohamed Babiker Musa, Youssry Mohamed Elsawy Ibrahim Aboelhassan, Olaleke Noah Olabode, Oumnia Bouaddi, Dawit Tesfagiorgis Mengesha, Abioye Sunday Micheal, Mohamed Abdul Kareem Adam Modber, Alhaji Umar Sow, Sara Gabrallah M. Kheir, Deborah Oluwaseun Shomuyiwa, Oso Tolutope Adebimpe, Emery Manirambona, Don Eliseo Lucero-Prisno

**Affiliations:** a Department of Medical Laboratory Science, Edo State University Uzairue, Benin; b Department of Medical Laboratory Science, Neuropsychiatric Hospital, Aro, Abeokuta; c Global Health Focus Africa, Abuja; d Obafemi Awolowo University Teaching Hospital Complex, Ile-Ife; e Faculty of Basic Medical Sciences, Department of Public Health, Adeleke University, Ede, Osun State; f Faculty of Pharmacy, University of Lagos, Lagos, Nigeria; g Faculty of Pharmacy, Omdurman Islamic University; h Faculty of Nursing Sciences, University of Khartoum; i The Global Fund-PMU-Sudan Federal Ministry of Health, Khartoum, Sudan; j Research Assistant, Research and development (R&D), Heliopolis University, Cairo, Egypt; k International School of Public Health, Mohammed VI University of Health Sciences, Casablanca; l Mohammed VI Center For Research and Innovation, Rabat, Morocco; m Saint Paul’s Hospital Millennium Medical College, Addis Ababa, Ethiopia; n College of Medicine and Allied Health Sciences, University of Sierra Leone, Freetown; o College of Medicine and Health Sciences, University of Rwanda, Kigali, Rwanda; p Research Unit, Global Health Focus, Bujumbura, Burundi; q Department of Global Health and Development, London School of Hygiene and Tropical Medicine, London, UK

**Keywords:** stigma, infectious diseases, Africa, social stigma, epidemiology

## Abstract

Stigma poses a significant barrier to accessing care, managing, and preventing infectious diseases in Africa. The authors conducted an extensive search across Scopus, PubMed, ScienceDirect, and Google Scholar to identify relevant English-language articles, with no constraints on publication dates, using the keywords “Stigma,” and “Infectious Disease,” in conjunction with “Africa.” This article explores the multifaceted nature of stigma associated with infectious diseases, highlighting its impact on healthcare access and public health outcomes. It delves into the current situation of infectious disease-related stigma in Africa, emphasizing the various diseases and contexts affected. The article identifies drivers of stigma, including negative attitudes, misinformation, and institutional practices, and discusses their role in perpetuating discrimination. Importantly, it provides recommendations for addressing infectious disease stigma in Africa through comprehensive strategies encompassing health education, contact-based interventions, professionalized counselling and peer support services, and community engagement. The article calls for collaboration among governments, healthcare organizations, NGOs, and community leaders to implement holistic strategies that prioritize inclusivity and stigma reduction. Ultimately, it underscores the urgent need to combat stigma to improve healthcare access and outcomes for individuals affected by infectious diseases in Africa.

## Introduction

Highlights

- Engaging marginalized groups is vital in addressing stigma in Africa.
- A multifaceted approach is recommended to mitigate stigma’s effects.
- Practical interventions are essential in alleviating the burden of stigma in Africa.

Stigma poses a growing challenge in Africa, hindering access to care for infectious diseases. Infected individuals hide their illnesses to avoid discrimination, impeding diagnosis and treatment and worsening the disease burden^1^. Stigma is defined as a mark of shame associated with a specific context, trait, or label, causing feelings of difference and exclusion, leading to social isolation and reduced self-esteem^2^. The resulting lack of social contact and self-worth can foster fear and otherness, culminating in stigma^1^.

Africa bears nearly 40% of the global burden of infectious and neglected tropical diseases, affecting about 400 million people, with ~185 000 deaths annually and 620 million disability-adjusted life years lost^3^. This does not account for the suffering and disability endured by over a billion individuals in impoverished regions^4^. The populations at risk in Africa include vulnerable groups like rural communities, urban slum dwellers, children, pregnant women, displaced populations, agricultural and rural workers, and indigenous communities^5^.

Stigmatization can represent the disease itself and the perception of being a carrier^6^. Stigma can be internalized, impacting self-perception and behaviour. Self-stigma refers to adverse actions taken against oneself or someone due to their infected status^7^, while perceived public stigma refers to the anticipation of discrimination. Disease-related stigma leads to fear-driven negative behaviours, attitudes, and judgments towards infected individuals^8^, hindering progress in disease control despite scientific advancements. Individuals with diseases like HIV/AIDS, ebola, tuberculosis, zika virus, Covid-19, and monkeypox are highly likely to experience stigma^1^.

The consequences include limited access to quality healthcare services, sustained transmission, and difficulties in containing the spread of infectious diseases^6^. Addressing stigma becomes imperative. In this study, we examine the role of stigma in disease outbreaks and propose strategies to mitigate its impact, offering a pathway to overcome disease outbreaks in Africa.

## Current situation of stigma and infectious diseases in Africa

In Africa, infectious diseases are accompanied by a formidable adversary: stigma. Stigma, the marginalization of individuals afflicted by diseases, leads to discrimination against them and their families^9^. While quantifying the exact extent of infectious disease-related stigma in Africa is challenging due to its diverse diseases and contexts, it is clear that stigma casts a significant and far-reaching shadow. Diseases such as HIV/AIDS, tuberculosis, and leprosy have been extensively documented as magnets for stigma^10,11^. Vulnerable populations, including key populations and adolescents, often face discrimination when seeking care^12^. Privacy breaches, differential treatment, and compromised counselling compound the problem^13^. Factors like gender, identity, poverty, and age contribute to HIV stigma in Kenya, with women and gender-diverse individuals experiencing discrimination when accessing clinical care^14^.

Recent outbreaks of diseases like Ebola, Covid-19, and monkeypox have exposed how stigma continues to hinder healthcare access, especially for vulnerable groups such as men who have sex with men^15^. In South Africa, a significant percentage of HIV-positive individuals experienced high levels of HIV-related stigmatizing attitudes, while stigma related to type 2 diabetes ranged from 49.6% in Ghana to 70% in Ethiopia^16,17^. Stigma was reported by Ebola survivors and their communities in DR Congo, Guinea, and Liberia^18^. A study from low- and middle-income countries found a prevalence of stigma at 37%^19^. Covid-19 has also ignited stigma against specific minority groups and foreigners. Even before the emergence of Covid-19, research consistently indicated that individuals living with HIV were more susceptible to stigma compared to those with other infectious diseases^20^. This stigma encompasses medical, moral, and social dimensions, perpetuating itself through interpersonal and intrapersonal channels. Moreover, individuals diagnosed with Covid-19 have faced heightened stigma, driven by increased public anxiety^21^. According to recent research, people with non-communicable diseases (NCDs) in Africa experience stigma and prejudice in a manner comparable to that experienced by people with infectious diseases, given the rising prevalence of NCDs across the world, which account for 80% of the mortality rate in Africa^22^. Numerous studies have shown that NCDs in Africa are also being stigmatized against, as evidenced by the perceived stigma suffered by people with NCDs ranging from 12 to 86% and the experienced stigma ranging from 5.6 to 68.5%^23,24^.

Many African nations have undertaken several efforts to address infectious disease stigma through public health campaigns, community engagement, education, healthcare professional training, and policy development^25,26^. These comprehensive strategies aim to destigmatize infectious diseases, raise public awareness, protect individuals’ rights, and ensure equitable healthcare access. Future plans involve intensifying awareness campaigns, integrating mental health support into primary care, enhancing data collection, fostering international collaboration, and reducing stigma in healthcare practices^27,28^. These efforts, geared towards a stigma-free future, reflect a steadfast commitment to tackling stigma and promoting awareness as integral components of public health initiatives across Africa^29^. Public-private partnerships addressing infectious diseases in Africa also incorporate stigma reduction, although to varying degrees, through initiatives such as public awareness campaigns and community engagement^30^. As these programs evolve, they forge partnerships among governments, NGOs, pharmaceutical firms, and the private sector, collectively working towards improved healthcare access and prevention measures^31,32^.

## Drivers of stigma in Africa

Stigma related to infectious diseases in Africa arises from negative attitudes, a lack of awareness, and institutional practices. Limited knowledge and misconceptions contribute to stigmatizing behaviours, even within healthcare settings. Fear of infection and fear of the individual or their behaviour also play a significant role^33^. Stigma is influenced by social and structural factors, including cultural and gender norms, as well as laws and policies. These factors shape the stigmatization process and impact Africa’s efforts to combat infectious diseases such as HIV, leprosy, tuberculosis, and others^34,35^.

Stigma within African communities is also driven by feelings of disgust and acts of marginalization towards individuals infected with infectious diseases. This perception stems from the belief that infectious diseases are primarily associated with disadvantaged backgrounds, seen as carrying a contagious and dirty agent^1,6^. In contrast, non-communicable diseases like cancer, diabetes, and hypertension, which lack an infectious agent, are often considered diseases of the affluent, despite receiving varied stigmatization recently. Infectious diseases are also perceived as easily transmissible through physical touch and social interaction^36^.

The societal interpretation of infectious diseases and the physical disfigurement associated with certain conditions, particularly neglected tropical diseases, contribute to stigma. Misinformed gossiping about specific contagious diseases, and exacerbates the burden of stigma among the infected population in Africa^15,25^. This leads to self-isolation and fear of stigmatization among marginalized groups, limiting their access to social and healthcare support services. Blaming individuals for not adhering to precautionary measures is also observed among vulnerable populations^37^.

## Recommendations for addressing infectious diseases stigma in Africa

Addressing stigma related to infectious diseases in Africa requires a comprehensive and effective approach. Proactively addressing and countering disease-related stigma during outbreaks is crucial for African governments, policymakers, and emergency response coordinators. To achieve this, a multifaceted strategy should be implemented, focusing on education, outreach, counselling, problem-solving, advocacy, and community engagement. These strategies collectively contribute to mitigating the negative impacts of stigma^6^.

### Health education, literacy and awareness

To tackle the issue of stigma related to infectious diseases in Africa, it is crucial for governments and community leaders to actively embrace educational programs and interventions aimed at countering stigma. These initiatives should focus on dispelling misinformation, challenging misconceptions, and correcting false beliefs that exist across the continent. Education campaigns, public lectures, and local and national awareness programs should be utilized to replace these myths and stereotypes with accurate information. Leveraging the power of social media platforms can facilitate reaching a larger audience, including individuals, groups, and communities^38^. By fostering understanding and promoting inclusivity, this comprehensive approach will effectively combat stigma throughout the continent^39^.

### Contact-based interventions

Building bridges and promoting acceptance will help overcome interpersonal division and facilitate healthy relationships and interactions between groups. One effective strategy is to create educational platforms where individuals who have successfully overcome stigmatized conditions can share their personal stories, obstacles, and triumphs with the broader public^38^. By presenting factual information derived from their own life experiences, these individuals can help dispel misconceptions and provide valuable insights. This approach not only breaks down barriers but also fosters safer and healthier relationships within communities. Furthermore, it promotes acceptance, inclusivity, and support for reintegration, thus making significant strides towards reducing stigma in Africa^18^.

### Enhancing support and professionalization of counselling and peer services

To address the stigma, rejection, and discrimination faced by stigmatized individuals when seeking help and treatment services, it is imperative to promote and establish counselling and peer support services across all sectors of healthcare, institutions, and communities. These services should be made accessible at the grassroots level, ensuring that no individual is left behind. By providing support and guidance, these services play a crucial role in reducing stigma, advancing human rights, and improving the overall quality of life for stigmatized individuals^40^.

Professionalizing these services is essential to enhance the effectiveness of behavioural health facilities throughout Africa. By establishing policies that prioritize mental processes, environmental factors, institutional actions, and socioeconomic influences, policymakers can create a comprehensive framework that positively influences the reduction of stigma. It is vital to incorporate stigma-related policies fully into healthcare systems to mitigate the detrimental effects of health-related stigma across all African communities^40,41^.

### Empowering communities

To effectively tackle the stigma surrounding infectious diseases in Africa, the government should actively encourage community members to serve as bridges between organizations and their respective communities. These community members play a vital role in effective communication, addressing public skepticism, and building trust by being relatable figures within the community. This approach fosters stronger relationships, actively reduces stigma, shame, and fear, and consequently promotes trust, inclusivity, and active community engagement, leading to more impactful efforts in stigma reduction.

Community meetings and engagements focused on marginalized groups can serve as pivotal platforms for addressing stigma in Africa. Facilitating open communication between public health officials and the public is crucial in any disease outbreak response^42^. Adopting a systematic approach to gathering valid and reliable information is also essential to moving forward. Working closely with community leaders and reputable groups can provide valuable insights into the specific challenges faced by stigmatized groups, complement public health efforts, and serve as vital channels for disseminating information to individuals who may not trust the government or other private sources^43^.

To ensure wide-reaching impact, it is imperative for African governments, legislators, healthcare organizations, international bodies, NGOs, and community leaders to join forces and collaboratively develop a holistic strategy. This strategy must encompass all recommended measures for stigma reduction and be implemented at every level of government in each African nation, leaving no community behind. The ultimate objective is to eradicate the stigma associated with infectious diseases, making inclusivity a top priority. Regular progress assessments and knowledge sharing among nations will further strengthen this collective effort to reduce stigma and foster understanding throughout Africa^29,45^.

## Conclusion

Stigma is a prevalent issue in African communities, which negatively impacts individuals affected by infectious diseases and their families. Tackling stigma requires a multifaceted approach across various sectors, including the community, workplace, education, healthcare, justice, and emergency. This involves fostering engagement, building trust, creating inclusivity, raising awareness, improving communication, implementing policy changes, and ensuring swift access to services. By engaging the community, promoting understanding, dispelling misconceptions, and implementing supportive policies, Africa can reduce the negative impacts of stigma and provide essential support to those affected by infectious diseases.

## Ethical approval

Approval from the ethical committee was not applicable.

## Consent

Consent has been obtained for this study.

## Source of funding

We declare that no funding was received for this work.

## Conflicts of interest disclosure

The authors declare no conflict of interest.

## Author contribution

P.O.O., O.O.J., M.B.M., Y.M.E.I.A., O.N.O. and O.B. conceived and designed the research, reviewed, analyzed, performed the research and wrote the paper. O.O.J., D.T.M., A.S.M., M.A.K.A.M., A.U.S., S.G.M.K., D.O.S., O.T.A. and D.E.L.P.III: interpreted the data, review-editing and proofread the paper. E.M. and D.E.L.P.III: interpreted the data, designed research, review-editing, supervision and proofread the paper.

## Research registration unique identifying number (UIN)

Not applicable.

## Guarantor

Okesanya Olalekan John.

## Provenance and peer review

Not commissioned.

## Data availability statement

Not applicable.

## Acknowledgements

None.
